# Peroxisomes in Liver Diseases: From Metabolite Quality Control to Inter-Organelle and Inter-Organ Signaling

**DOI:** 10.3390/biom16060895

**Published:** 2026-06-17

**Authors:** Carolina Hogerty, Yantao Zhao, Weiran Wang, Steven A. Weinman, Wei Zhong

**Affiliations:** 1 Department of Internal Medicine, University of Kansas Medical Center, Kansas City, KS 66160, USA; 2 The Liver Center, University of Kansas Medical Center, Kansas City, KS 66160, USA; 3 Department of Pharmacology, Toxicology and Therapeutics, University of Kansas Medical Center, Kansas City, KS 66160, USA

**Keywords:** peroxisome, liver disease, lipid metabolism, redox signaling, organelle crosstalk

## Abstract

Peroxisomes are essential metabolic organelles that support core aspects of cellular homeostasis. In the hepatocytes, peroxisomes govern key aspects of cellular homeostasis, including processing lipid substrates that are inadequately handled by mitochondria, controlling hydrogen peroxide metabolism, and regulating bile acid synthesis. Increasing evidence indicates that these organelles are not merely auxiliary metabolic compartments but active contributors to the development and progression of liver disease. Dynamic alterations in peroxisomal proteins and function are being noted. Across metabolic dysfunction-associated steatotic liver disease, alcohol-associated liver disease, cholestatic disorders, fibrosis, and hepatocellular carcinoma, peroxisomes undergo remodeling that shows a change from adaptive reactions to maladaptive states. These changes perturb signaling pathways that regulate inflammation, stress responses, and cell fate. In addition, because peroxisomes operate within an interconnected organelle network, their dysfunction propagates to mitochondria, endoplasmic reticulum, and other cellular systems, amplifying metabolic and cellular stress. This review summarizes current understanding of how peroxisomal pathways contribute to liver disease, highlighting mechanisms involving lipid accumulation, oxidative stress, and disrupted organelle crosstalk. How peroxisome-dependent control of circulating metabolites links hepatic injury to extrahepatic organ systems is further discussed. At the end, emerging therapeutic strategies for liver disease targeting peroxisomal pathways are discussed. Together, the emerging understanding of peroxisomal remodeling, metabolic regulation, organelle crosstalk, and inter-organ communication positions peroxisomes as active and dynamic regulators of liver disease and potential targets for therapeutic intervention.

## 1. Introduction

Running under continuous metabolic stress, the liver keeps processing dietary lipids, xenobiotics, and endogenous metabolites and maintains redox balance and systemic metabolic homeostasis [1,2]. This function relies on coordinated activity among intracellular organelles that regulate lipid metabolism, detoxification, and stress reactions [3,4,5]. In addition, peroxisomes have conventionally been mainly in charge of very long-chain fatty acid β-oxidation and reactive oxygen species detoxification as auxiliary metabolic compartments. Nevertheless, their wider effect on hepatic physiology and disease is underestimated by this classical perspective. Profound liver dysfunction frequently accompanies Zellweger spectrum disorders and other inherited peroxisomal disorders [6], offering convincing clinical proof that peroxisomes are indispensable for keeping hepatic metabolic homeostasis.

Peroxisomes are uniquely positioned to regulate metabolite composition and quality of intracellular lipid pools [2,7,8]. By processing lipid substrates that are inadequately handled by mitochondria, and by catalyzing core steps in bile acid and ether lipid biosynthesis, peroxisomes extend the metabolic capacity of hepatocytes. In the meantime, hydrogen peroxide is produced as a byproduct of lipid metabolism, which places these organelles at the center of intracellular redox networks. Instead of only acting as detoxification sites, peroxisomes work as redox signaling platforms affecting inflammatory reactions, stress adaptation, and cellular homeostasis. Emerging evidence further indicates that peroxisomes run in a comprehensive organelle network [3,4,5]. With functional coupling with mitochondria, endoplasmic reticulum (ER), lipid droplets, and lysosomes, cooperative regulation of lipid flux, membrane composition, and organelle quality is enabled. Through these interactions, peroxisomes contribute to the regulation of hepatocellular metabolism by extending beyond their intracellular enzymatic activities. This interaction also indicates that disruption of peroxisomal function can propagate across organelle systems, amplifying metabolic imbalance and cellular stress [9].

Chronic liver diseases progress through a continuum of hepatocellular injury, inflammation, and fibrosis, often resulting in hepatocellular carcinoma. Continuous metabolic stress, oxidative injury, and inflammatory signaling drive the progression [10]. In many liver diseases, peroxisomal dysfunction is viewed more as a disease driver instead of only a passive consequence of injury [11,12,13]. A wide spectrum of conditions, such as metabolic dysfunction-associated steatotic liver disease (MASLD) [12,14], alcohol-associated liver disease (ALD) [15], cholestatic disorders [16], fibrosis [17], and hepatocellular carcinoma (HCC) [18,19], show changes in peroxisomal abundance, enzymatic activity, and metabolic output. Under these pathological conditions, peroxisomes show dynamic remodeling, which indicates that disease progression may show a transformation from adaptive reactions to maladaptive states with the characteristics such as dysregulated redox signaling, impaired lipid handling, and changed metabolite profiles.

This review focuses on peroxisomes in liver disease from a hepatocyte-oriented view, highlighting how redox balance, lipid metabolism, and inter-organelle communication are regulated by peroxisomal pathways. How peroxisome-dependent variations in metabolite composition extend beyond the liver to affect systemic physiology is further explored. In the end, novel therapeutic measures and translational difficulties are discussed. Jointly, these understandings back a model in which peroxisomes serve as metabolite quality control hubs, whose dysfunction makes contributions to initiating and progressing liver disease.

## 2. Liver-Specific Biology of Peroxisomes: Metabolic Specialization and Signaling Functions

**Figure 1:**
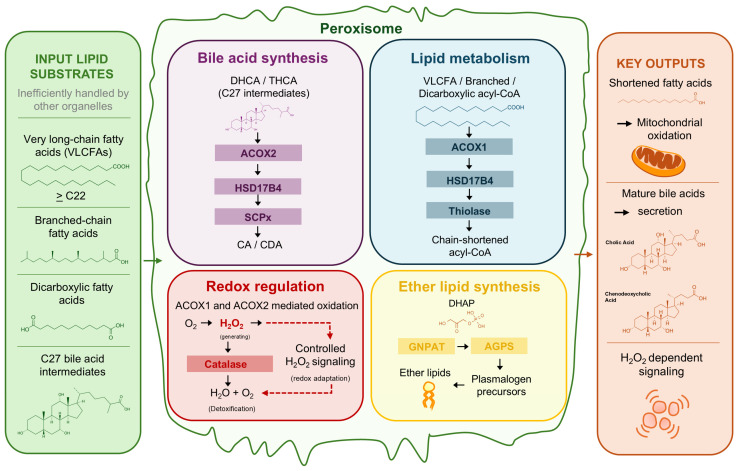
Peroxisomes as a metabolite quality control hub in hepatocytes. Peroxisomes selectively process lipid substrates that are inefficiently handled by other organelles, including very long-chain, branched-chain, and dicarboxylic fatty acids, as well as C27 bile acid intermediates. Through these pathways, they generate shortened fatty acids for mitochondrial β-oxidation, produce mature bile acids for secretion, and regulate hydrogen peroxide-dependent redox signaling. Rather than controlling bulk metabolic flux, peroxisomes dynamically maintain metabolite quality.

As single-membrane-bound organelles, peroxisomes are highly enriched in hepatocytes [20], reflecting the central effect of the liver on lipid metabolism and detoxification (Figure 1). Unlike mitochondria, peroxisomes do not have an endosymbiotic origin and are short of their own genome. On the contrary, they are thought to arise mainly from the ER and reply completely on post-translational import of nuclear-encoded proteins for their biogenesis and function [3]. Hence, their metabolic functions are closely related to cellular signaling and transcriptional programs that control organelle abundance and activity. Detailed biochemical pathways involved in peroxisomal β-oxidation, bile acid chain shortening, and hydrogen peroxide metabolism have been comprehensively reviewed elsewhere [21,22,23,24,25], and only core aspects related to liver physiology and disease are emphasized here.

### 2.1. Lipid Metabolism and Metabolic Partitioning

A defining feature of hepatic peroxisomes is their capacity of processing lipid substrates that are inadequately handled by mitochondria, which expands the metabolic capacity of the cell [9,26]. These encompass very long-chain fatty acids, branched-chain fatty acids, and dicarboxylic fatty acids produced via ω-oxidation. These substrates are shortened by peroxisomal β-oxidation and produce intermediates that can subsequently be transferred to mitochondria for complete oxidation.

By comparing with mitochondrial β-oxidation, peroxisomal oxidation is not coupled with ATP manufacturing [26]. On the contrary, the direct transfer of electrons to molecular oxygen generates hydrogen peroxide. Therefore, peroxisomes make minimal contributions to cellular energy manufacturing but exert a key effect on regulating the composition of intracellular lipid pools. It is especially significant in hepatocytes, where volatility in lipid availability demands quick and selective processing of various lipid species. In virtue of this substrate specialization, peroxisomes serve as a metabolic filtering system, which stops accumulating structurally complicated lipids that can damage membrane integrity, signaling pathways, and organelle function. This effect becomes particularly related under circumstances of lipid overload, where peroxisomal ability is necessary for maintaining metabolic balance [12].

### 2.2. Bile Acid Synthesis and Cholesterol Disposal

Peroxisomes are important in bile acid synthesis, a pathway that stands for the main route for cholesterol removal in the liver [27]. Despite early procedures in bile acid synthesis in the ER and mitochondria, the terminal responses that convert C27 bile acid intermediates into mature C24 bile acids through β-oxidation of the side chain are catalyzed by peroxisomes [28]. It is necessary for generating bile acids effectively conjugated and secreted into bile. Impairing peroxisomal function results in accumulating bile acid intermediates and changed bile acid composition, which highlights the significance of peroxisomes in keeping bile acid homeostasis.

In addition to their effect on cholesterol disposal, bile acids control metabolic pathways, inflammation, and enterohepatic communication as signaling molecules [29]. Therefore, peroxisomal control over bile acid composition shows influences that exceed hepatocytes. The balance of bile acid species can be changed by changes in peroxisomal function, which potentially modifies signaling in virtue of farnesoid X receptor (FXR), influencing processes including lipid metabolism, glucose homeostasis, and gut–liver communications.

### 2.3. Ether Lipid Biosynthesis and Membrane Organization

The biosynthesis of ether phospholipids, such as plasmalogens [30], is initiated by peroxisomes through reactions that happen exclusively in the organelle. As a subclass of glycerophospholipids, plasmalogens are differentiated by a vinyl ether linkage at the sn-1 position of the glycerol backbone instead of the typical ester bond discovered in the traditional phospholipids. Ether lipid intermediates produced in peroxisomes are later processed in the ER, so as to generate mature ether lipids included in cellular membranes. Ether lipids make contributions to membrane framework, fluidity, and the organization of lipid microdomains, affecting processes including vesicle trafficking and signal transduction. In addition, plasmalogens have been important in oxidative stress protection because their vinyl ether bond can serve as a preferential target for reactive oxygen species [31,32].

### 2.4. Redox Regulation

Peroxisomal metabolism is tightly associated with reactive oxygen species manufacturing and detoxification [8,33,34,35]. During fatty acid oxidation and other oxidative reactions, peroxisomal enzymes produce hydrogen peroxide, which is later decomposed by catalase and other antioxidant systems in the organelle. This dual ability to generate and remove reactive oxygen species places peroxisomes at the center of intracellular redox control.

Particularly, hydrogen peroxide works as a signaling molecule that can regulate protein activity via reversible oxidation of cysteine residues. Through controlled manufacturing and diffusion of hydrogen peroxide, peroxisomes contribute to the regulation of signaling pathways involved in metabolism, stress reactions, and inflammation. Tight coordination between peroxisomal oxidases and antioxidant systems is required by maintaining redox homeostasis [35]. Disruption of this balance can result in excessive accumulation of reactive oxygen species, which promotes oxidative stress, lipid peroxidation, and activation of inflammatory pathways. On the contrary, adaptive responses to metabolic stress may be impaired by inadequate reactive oxygen species signaling. Significantly, peroxisomal redox signaling is closely related to other organelles. For example, mitochondria stand for a main source of reactive oxygen species, and crosstalk between peroxisomes and mitochondria makes contributions to coordinating cellular redox tone [3]. Section 3 discusses these interactions and broader organelle crosstalk in more detail.

## 3. Peroxisomes as an Adaptive Organelle Network: Biogenesis, Turnover, and Crosstalk

### 3.1. Biogenesis and Protein Import: Maintaining Functional Capacity

Peroxisomes depend entirely on nuclear-encoded proteins that are synthesized in the cytosol and imported post-translationally [3]. This process is mediated by a conserved set of peroxins (PEX proteins) that coordinate membrane assembly, matrix protein import, and organelle maintenance [21,22,23]. Matrix proteins carrying peroxisomal targeting signals are recognized by cytosolic receptors and delivered to the peroxisomal membrane, where they are translocated into the organelle and the receptors are recycled in an ATP-dependent manner.

Peroxisomes can arise through both de novo biogenesis from the ER and growth and division of pre-existing organelles [36]. In hepatocytes with high and fluctuated metabolic need, quick modification of peroxisomal capacity is backed by this dual system. Protein import and organelle assembly are impaired by disrupting peroxin function, resulting in a loss of metabolic capacity despite existing peroxisome-like frameworks. Instead of a static compartment, a balance between biogenesis, protein import efficiency, and functional enzyme content is shown by the peroxisomal population in hepatocytes. This dynamic control is key to keeping the metabolic and redox effects of peroxisomes under both physiological and stress circumstances.

### 3.2. Peroxisomal Plasticity: Proliferation, Turnover, and Disease-Stage Remodeling

Their enrichment and activity in reaction to metabolic cues are modified by peroxisomes, highly plastic organelles. In the liver, lipid overload, fasting, and activation of nuclear receptors (i.e., peroxisome proliferator-activated receptor alpha/PPARα) can induce peroxisome proliferation [9,37,38], leading to grown capacity for fatty acid oxidation and redox buffering. With this adaptive extension, hepatocytes are allowed to react to transient metabolic stress through improving lipid processing and restricting accumulation of toxic intermediates. The elimination of damaged or excess peroxisomes through selective autophagy, or pexophagy, is equally significant [38,39]. This quality control process prevents the persistence of dysfunctional organelles that could contribute to oxidative stress and metabolic imbalance. Jointly, proliferation and turnover keep a functionally capable peroxisomal pool.

Peroxisomal dynamics frequently change from adaptive to maladaptive states in liver disease. Grown peroxisome enrichment may be related to early stages of metabolic or toxic injury, showing a compensatory reaction. Nevertheless, with disease progression, this reaction can be inadequate or dysregulated, resulting in decreased functional capacity despite unvaried or even grown organelles. This transformation emphasizes a significant theory: peroxisomal dysfunction in liver disease is often a failure of adaptation instead of a simple loss of organelles.

### 3.3. Organelle Crosstalk: Integrating Metabolism and Stress Responses

#### 3.3.1. Peroxisome–Mitochondria Interactions

**Figure 2:**
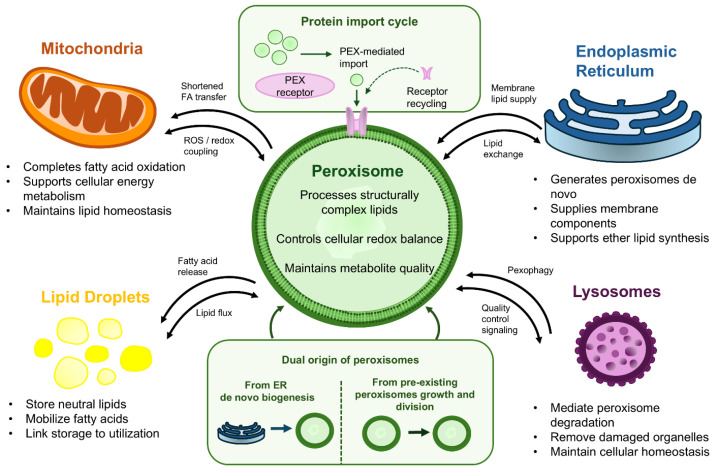
Peroxisomes as an inter-organelle network hub in hepatocytes. Peroxisomes function within a dynamic organelle network, interacting with mitochondria, endoplasmic reticulum, lipid droplets, and lysosomes to coordinate lipid metabolism, redox balance, and organelle turnover. These interactions integrate metabolic flux with organelle function.

Peroxisomes run in an interconnected network of organelles jointly controlling hepatic metabolism (Figure 2) [3,4,5]. Among them, the association between peroxisomes and mitochondria is especially important [40,41]. These organelles collaborate in fatty acid oxidation, with peroxisomes starting the breakdown of very long-chain and structurally complicated fatty acids and mitochondria finishing their oxidation. Efficient transfer of metabolic intermediates among these compartments is necessary for maintaining lipid homeostasis. In addition to metabolic coupling, peroxisomes and mitochondria are closely associated in virtue of redox control [42,43]. Reactive oxygen species are produced by both organelles, and their antioxidant systems must work in a coordinated way to keep cellular redox balance. Hence, disruption of peroxisomal redox control can affect mitochondrial function, resulting in grown oxidative stress and impaired energy metabolism. The presence of physical contact sites between peroxisomes and mitochondria is indicated by new proof [40], which facilitates the exchange of metabolites and signaling molecules. With these interplays, quick adaptation to variations in metabolic demand is enabled, and contributions to the integration of lipid metabolism with cellular energy status are made.

#### 3.3.2. Peroxisome-Endoplasmic Reticulum Interactions

Close functional and structural associations with the ER, which acts as a core platform for peroxisome biogenesis and lipid metabolism, are maintained by peroxisomes. Peroxisomes can arise partially from the ER by de novo formation, where pre-peroxisomal vesicles bud from ER and mature into functional peroxisomes [44,45]. In addition, direct physical contact sites driving the exchange of lipids and signaling molecules are formed by peroxisomes and ER. These interplays support coordinated control of lipid metabolism, such as ether phospholipid synthesis spanning both organelles. Membrane components and lipid precursors are provided by the ER, while lipid composition and downstream processing are shaped by peroxisomal metabolism. In virtue of biogenetic linkage and physical coupling, hepatocytes integrate lipid synthesis, processing, and stress signaling by the peroxisome-ER axis, and its disruption may cause metabolic imbalance in liver disease.

#### 3.3.3. Lipid Droplets, Lysosomes, and Organelle Quality Control

In addition, peroxisomes make interaction with lipid droplets and lysosomes [9,46,47], which further realize integration between lipid metabolism and cellular quality control systems. As storage depots for neutral lipids, lipid droplets can be mobilized and sent to peroxisomes for oxidation. The interplay links lipid storage with metabolic adoption, especially under circumstances of grown lipid flux. Lysosomes allow selective deterioration of damaged or excess peroxisomes by making contributions to peroxisomal turnover through pexophagy [39]. This process is key to keeping organelle integrity and stopping accumulation of dysfunctional peroxisomes.

## 4. Peroxisomes Across the Liver Disease Spectrum

**Figure 3:**
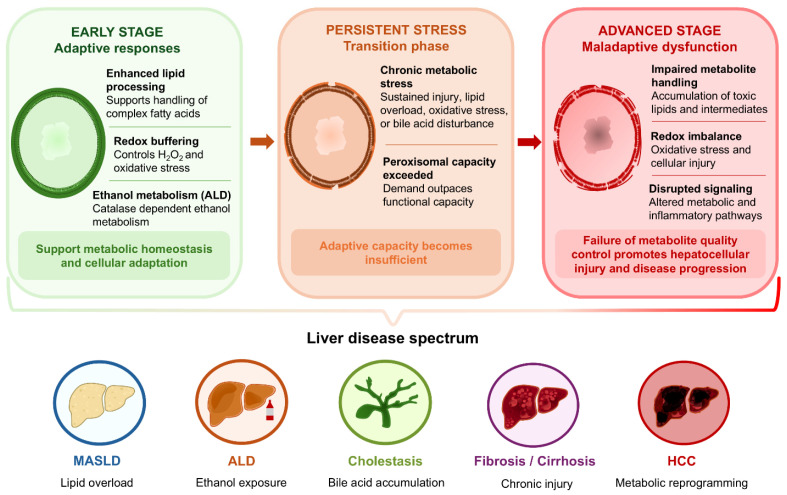
Peroxisomes in liver disease: adaptive responses and maladaptive dysfunction. Peroxisomes experience dynamic variations across liver diseases. In early stages, they back adaptation in virtue of improved lipid oxidation, redox buffering, and, in ALD, catalase-dependent ethanol metabolism. With continuous stress, peroxisomal function becomes impaired, resulting in accumulation of toxic metabolites, redox imbalance, and disrupted signaling. This transition makes contributions to disease progression across MASLD, ALD, cholestasis, fibrosis, and hepatocellular carcinoma, showing failure of metabolite quality control.

Peroxisomal function is changed in a dynamic way across major liver diseases, where it makes contributions to disease progression in virtue of roles in lipid metabolism, redox balance, and inter-organelle communication. Despite the variation in specific drivers among disease backgrounds, an ordinary theme is the transformation from adaptive peroxisomal reactions backing metabolic homeostasis to maladaptive states. How these processes manifest across main liver diseases is emphasized below with attention to hepatocyte-centered systems (Figure 3).

### 4.1. Metabolic Dysfunction–Associated Steatotic Liver Disease (MASLD)

The exposure of hepatocytes to chronic lipid overload generates a continuous demand for fatty acid oxidation in MASLD [48,49]. As recent reviews of peroxisome dysfunction in MASLD have highlighted, impaired peroxisomal lipid handling, redox imbalance, and defective organelle crosstalk are all related to MASLD progression [12]. Peroxisomes contribute to an adaptive response by enhancing the processing of very long-chain and structurally complex fatty acids, thereby helping restrain the accumulation of lipotoxic intermediates and maintain metabolic flexibility [50,51]. This adaptive role is supported by studies demonstrating that peroxisomal β-oxidation pathways, particularly those mediated by acyl-CoA oxidase 1 (ACOX1), are important regulators of lipid metabolism and inflammatory responses [52,53,54]. However, increasing evidence suggests that the effects of ACOX1 may be context dependent. In a recent MASLD study, FXR-driven ACOX1 activation was associated with increased lipotoxicity during obeticholic acid treatment, whereas pharmacological inhibition of ACOX1 enhanced the efficacy of low-dose obeticholic acid and reduced hepatic steatosis and inflammatory signaling [55]. Emerging evidence further indicates that peroxisome biogenesis and peroxisomal protein networks are altered in MASLD. Hepatocyte-specific deletion of the peroxin PEX16, a key regulator of peroxisome membrane assembly, results in loss of functional peroxisomes and marked alterations in hepatic lipid metabolism under high-fat diet conditions, providing direct evidence that intact peroxisome biogenesis is required to maintain lipid homeostasis [56]. Consistent with this, expression of multiple peroxins, including PEX3, PEX5, and PEX13, is reduced in high fat diet-fed mice, and pharmacologic activation of PPARα restores peroxisomal “fitness” and improves hepatic steatosis [50]. In addition, with the persistence of lipid stress, peroxisomal ability may be inadequate regarding metabolic need. In this context, failure to properly process specific lipid species can damage signaling pathways and worsen lipotoxic stress while changing membrane composition. In the meantime, hydrogen peroxide production may be increased by growing dependence on peroxisomal β-oxidation. In case of insufficient antioxidant buffering, oxidative stress is increased, which promotes inflammatory signaling and transformation from simple steatosis to steatohepatitis [12]. Hence, hepatocellular injury is caused by peroxisomes change from an initially adaptive system buffering lipid overload to a maladaptive state where impaired metabolite handling and redox imbalance.

Beyond alterations in lipid metabolism, peroxisomal dysfunction may contribute to MASLD through disruption of inter-organelle communication networks. As discussed in Section 3.3, peroxisomes maintain dynamic interactions with multiple intracellular organelles. Peroxisomal defects can impair mitochondrial function, promote ER stress, alter lysosomal homeostasis, and disrupt cellular redox balance, thereby amplifying metabolic and inflammatory stress within hepatocytes. This disruption of peroxisome-related organelle crosstalk may represent an additional mechanism linking peroxisomal dysfunction to disease development.

The strongest evidence in MASLD remains hepatocyte-centered, but non-parenchymal cells likely modulate this process. Kupffer cells and monocyte-derived macrophages are now recognized as major drivers of inflammation and fibrosis in MASLD/MASH (metabolic dysfunction-associated steatohepatitis), and macrophage activation is tightly coupled to lipid handling and redox metabolism. Broader work on macrophage peroxisomes supports roles in inflammatory regulation, oxidative stress control, and lipid mediator metabolism [57,58], all of which are relevant to the pathogenesis of steatotic liver. Moreover, earlier work showed that Kupffer-cell–derived IL-1β can suppress hepatocyte PPARα signaling and promote steatosis [59], highlighting a hepatocyte-macrophage link that indirectly regulates peroxisome-related metabolic dysfunction. Hepatic stellate cells represent another relevant non-parenchymal population influenced by peroxisome-related pathways. Recent work with the pan-PPAR agonist lanifibranor showed suppression of fibrotic and inflammatory programs in human stellate cells and multicellular MASH models [60]. However, these findings should not be overinterpreted as evidence that stellate-cell peroxisomal dysfunction is a primary driver of MASLD. At present, the most defensible conclusion is that hepatocytes are the principal cell type in which peroxisomes are driving MASLD development, while macrophages and stellate cells represent important sites of peroxisomal involvement that need further exploration.

### 4.2. Alcohol-Associated Liver Disease (ALD)

Alcohol-associated liver disease is driven by the metabolic and redox consequences of ethanol exposure, including altered lipid handling, redox imbalance, and organelle stress [61]. In this context, peroxisomes are involved in some systems that are widely related across liver diseases, such as fatty acid metabolism, reactive oxygen species detoxification, and cooperation with mitochondria and ER. Peroxisomal β-oxidation may partially compensate when ethanol-induced decline in NAD^+^/NADH ratio suppresses mitochondrial fatty acid oxidation, thereby assisting in limiting the accumulation of structurally complicated lipid species [2,11]. In the meantime, peroxisomes make contributions to hydrogen peroxide metabolism and redox signaling [62], which places them at the center of oxidative stress reactions in alcohol-exposed hepatocytes. In addition, because peroxisomes are necessary for bile acid maturation, metabolic signaling in the injured liver may be further altered by disrupting peroxisomal function.

A characteristic that differentiates peroxisomes in ALD is their direct participation in ethanol metabolism. Catalase within peroxisomes can oxidize ethanol to acetaldehyde using hydrogen peroxide in the classic peroxidatic reaction [63,64]. Although this pathway makes less contributions to total ethanol metabolism, it presents a physiologically related alternative route that is tightly associated with peroxisomal redox activity [65,66]. A functional coupling between peroxisomal lipid metabolism and ethanol clearance is further supported by recent research [67,68,69]. Catalase-dependent ethanol metabolism is enhanced by activating PPARα, whereas this effect is reduced by inhibiting peroxisomal β-oxidation or catalase-deficiency [68]. In the associated work, activation of the PPARα-catalase axis promoted alcohol clearance, reduced reliance on CYP2E1-mediated ethanol metabolism, and ameliorated alcohol-induced liver injury, while alcohol metabolism and worsened injury were impaired by catalase deficiency [69]. The idea that intact peroxisomal activity is mainly helpful in ALD is backed by these outcomes.

Nevertheless, this adaptive effect comes with a cost. Hydrogen peroxide flux is related to peroxisomal β-oxidation and catalase-dependent ethanol oxidation. With exposure to chronic alcohol, antioxidant defenses can be overwhelmed by combined reactive oxygen species (ROS) generation from peroxisomes and mitochondria, resulting in lipid peroxidation, oxidative stress, and hepatocellular injury [62]. According to novel proof, there are dynamic peroxisomal reactions in ALD, with an early growth in functional peroxisomes during initial injury that is not continuous in advanced fibrotic or cirrhotic phases [15]. With a decline in peroxisomal capacity, hepatocytes become less capable of coordinating fatty acid oxidation, redox buffering, and metabolite processing. With this loss of adaptive function, peroxisomes may be changed from protective metabolic regulating agents to contributors to disease progression.

Hence, it appears that peroxisomal remodeling observes a transformation from adaptive compensation to maladaptive dysfunction in ALD. Early during ethanol exposure, peroxisomal activity backs hepatocyte adaptation by driving fatty acid oxidation and catalase-dependent ethanol metabolism. Nevertheless, with persistent injury, reducing peroxisomal function and impaired organelle coordination make contributions to metabolic dysregulation, oxidative stress, and progression of liver injury.

### 4.3. Cholestatic Liver Disease and Bile Acid-Driven Injury

In contrast to MASLD and ALD, where lipid overload and redox imbalance dominate, cholestatic liver diseases are characterized by intrahepatic accumulation of bile acids and progressive hepatocellular injury. Peroxisomes are important in this process because they catalyze the final β-oxidation–dependent shortening of C27 bile acid intermediates to mature C24 bile acids, a step necessary for normal bile acid conjugation and secretion [27,70].

In case of disrupted peroxisomal bile acid metabolism, C27 intermediates accumulate and make direct contributions to liver injury. This is supported by human genetic disorders influencing peroxisomal bile acid handling. Loss of ACOX2, which catalyzes the first step of peroxisomal side-chain shortening of bile acid intermediates, causes accumulation of toxic C27 bile acids and is related to persistent hypertransaminasemia and liver fibrosis [71,72]. Similarly, serious liver disease with striking accumulation of these metabolites is caused by ABCD3 deficiency, which impairs transport of C27 bile acid intermediates into peroxisomes [73]. These discoveries are mechanistically significant because C27 bile acid intermediates are relatively hydrophobic and appear to be toxic in peroxisomal disorders [13]. According to prior research, these intermediates impair mitochondrial function and make contributions to the liver disease related to peroxisomal defects [11,74]. Significantly, according to in vivo research, peroxisomal dysfunction changes bile acid homeostasis at various levels. In peroxisome-deficient PEX2 Zellweger mice, bile acid metabolism is seriously damaged, resulting in hepatic injury and changed expression of bile acid transporters. Remarkably, bile acid treatment changed bile acid transport while partially correcting hepatic abnormalities, suggesting that the composition of the bile acid pool can affect disease severity even in the context of peroxisomal deficiency [16]. Hence, peroxisomal dysfunction in cholestatic contexts can drive injury in virtue of impaired bile acid maturation and accumulation of hepatotoxic intermediates.

Bile acid signaling may also be shaped by peroxisomal defects. Because activation of receptors including FXR and TGR5 (Takeda G protein-coupled receptor 5) is determined by bile acid composition, feedback control of bile acid synthesis and metabolic and inflammatory signaling can be disrupted by changed bile acid pools [75,76,77]. Hence, peroxisomes affect the detoxification and secretion of bile acids and the signaling pathways that regulate bile acid homeostasis.

### 4.4. Fibrosis and Cirrhosis

Fibrosis and cirrhosis arise from persistent liver injury and show the cumulative results of hepatocyte stress, inflammation, and extracellular matrix deposition. The contribution of peroxisomes is best treated as hepatocyte-centered and indirect, instead of as a main driver of fibrogenesis. This link is backed by human peroxisomal disorders, as chronic liver disease, including fibrosis, is a recognized characteristic of peroxisomal dysfunction [11,78]. At the cellular level, peroxisomes affect pathways relevant to chronic liver injury, including lipid metabolism, redox balance, and bile acid homeostasis. Mitochondrial and ER homeostasis in hepatocytes can be perturbed by disrupting peroxisomal function, suggesting the propagation of peroxisomal defects across organelle systems. Although these variations conform to circumstances that drive chronic injury, direct proof linking peroxisomal dysfunction to fibrogenesis is still restricted.

Hepatic stellate cells finally execute fibrosis, and PPAR-directed pathways offer the clearest link between peroxisome-associated biology and fibrogenesis. In particular, PPARγ signaling helps maintain stellate cell quiescence, whereas loss of this program favors fibrogenesis [79,80]. Early mechanistic studies showed that restoring PPARγ activity suppresses stellate cell activation and attenuates fibrosis, and more recent work has confirmed that pharmacologic PPARγ activation ameliorates liver fibrosis in vivo while dampening fibrogenic programs in stellate cells [81]. Recent data further identify an adiponectin–PPARγ axis in hepatic stellate cells as an important regulator of fibrosis progression [82]. These studies primarily support an antifibrotic role for PPAR signaling itself rather than demonstrating that peroxisome proliferation or peroxisomal remodeling directly mediates fibrosis resolution.

These findings support an antifibrotic role for PPAR signaling and establish a functional link between peroxisome-associated metabolic pathways and fibrogenesis. However, current evidence suggests that enhanced peroxisome-associated metabolic activity and reduced fibrosis are parallel consequences of PPAR activation rather than evidence that peroxisomal remodeling itself directly mediates fibrosis resolution.

An additional layer of support comes from studies of fibrogenic signaling itself. TGF-β1 (transforming growth factor beta 1) has been shown to suppress peroxisomal genes and proteins through SMAD (suppressor of mothers against decapentaplegic)-dependent mechanisms [83], suggesting that fibrosis-promoting pathways may further impair peroxisomal capacity during chronic liver injury.

Taken together, current evidence supports a model in which peroxisomes influence fibrosis primarily by shaping the hepatocyte injury environment, whereas stellate cell activation and matrix deposition are governed by broader fibrogenic signaling networks. Direct mechanistic roles of peroxisomes in fibrogenesis remain to be defined.

### 4.5. Hepatocellular Carcinoma (HCC)

Hepatocellular carcinoma (HCC) arises in the setting of chronic liver injury and is characterized by profound metabolic reprogramming and altered redox homeostasis. Peroxisomes play a more significant role in tumor biology [18], although it appears that their effect is context-dependent and more complicated than in steatotic or cholestatic liver disease. According to human research, peroxisomal structure and protein expression are altered in liver tumors [19,84,85,86]. According to morphometric and cytochemical analyses, hepatocellular tumors show decreased catalase staining and obvious peroxisomal abnormalities compared with non-tumor liver [85], while later immunohistochemical work displayed reduced expression of catalase and some peroxisomal β-oxidation enzymes across human hepatic neoplasms, with the most obvious declines in poorly differentiated tumors [86]. According to these outcomes, hepatocellular transformation is accompanied by loss of normal peroxisomal identity.

According to functional research, peroxisomal pathways affect HCC mainly through metabolic and redox control. The peroxisomal β-oxidation enzyme ACOX1 has appeared as a core node in this process. In HCC models, its activity is restrained by sirtuin 5 (SIRT5)-mediated desuccinylation of ACOX1, restricting peroxisome-derived oxidative stress. Loss of SIRT5 grows ACOX1 activity, improves oxidative damage, and drives tumor development [87]. According to this, disruption of peroxisome function in liver cancer cells reduces cell viability while inducing metabolic stress and inhibiting mTOR (mechanistic target of rapamycin) signaling [88], indicating that several tumor cells remain reliant on intact peroxisomal metabolism for survival. Jointly, according to these studies, peroxisomes actively control tumor cell fitness by reaching a balance between lipid metabolism and redox homeostasis.

At the signaling level, a well-characterized link between peroxisomes and hepatocarcinogenesis is offered by PPARα [89]. Although liver tumors in rodents are induced by chronic PPARα activation, this reaction is species-specific and not directly applicable to humans [90,91,92]. On the contrary, according to mouse research on chemically induced HCC, PPARα deficiency increased tumor susceptibility, while ectopic PPARα expression in HCC cells drove apoptosis by inhibiting NF-κB (nuclear factor kappa-light-chain-enhancer of activated B) signaling while suppressing proliferation [93]. According to these outcomes, PPARα can work as a tumor suppressive pathway instead of a carcinogenic driver in existing liver cancer models related to human disease. Peroxisomes may also make contributions to tumor progression in virtue of context-specific signaling systems. The invasive ability of hepatocellular carcinoma cells is enhanced by peroxisome-localized HBx in hepatitis B virus-associated HCC models, offering a direct instance of how malignant action can be modulated by peroxisome-related signaling [94]. As this discovery emphasizes, besides metabolic functions, peroxisomes can act as platforms for signaling events that affect tumor progression.

Generally, a model where peroxisomes affect HCC in virtue of changes in peroxisomal protein expression, dysregulated metabolic and redox regulation, and PPARα-dependent signaling pathways is backed by present proof. Rather than acting as consistent oncogenic drivers, peroxisomes adjust the tumor context in manner that may either suppress or support tumor progression depending on context.

## 5. Beyond the Liver: Peroxisome-Dependent Liver–Organ Communication

**Figure 4:**
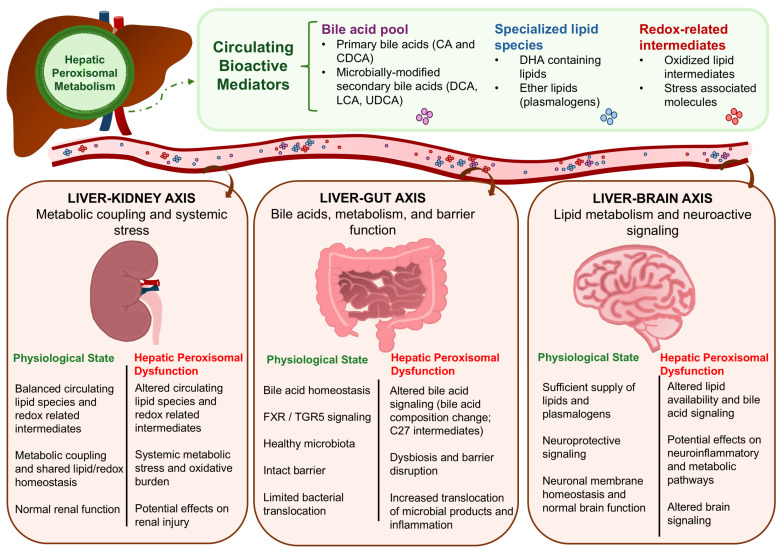
Peroxisome-dependent liver–organ communication. Hepatic peroxisomes regulate systemic metabolic homeostasis through the production and processing of bile acids, lipid species, and redox-associated metabolites. These metabolites mediate communication between the liver and peripheral organs, including the intestine, kidney, and brain. Disruption of these communication networks may contribute to liver disease progression and extrahepatic complications.

Although peroxisomes are important in hepatocytes, the impact of hepatic peroxisomes exceeds the liver by regulating circulating metabolites and signaling molecules. Hepatic peroxisomes make contributions to exchange between the liver and peripheral organs through forming the composition of lipid species, bile acids, and redox-associated intermediates. As illustrated in Figure 4, this inter-organ signaling is treated more as a significant ingredient of systemic metabolic control and liver disease progression.

### 5.1. Role of Peroxisomes in the Liver–Gut Axis: Bile Acids, Metabolism, and Barrier Function

The liver–gut axis stands for a core pathway through which extrahepatic physiology is affected by peroxisomal metabolism, with reciprocal feedback roles in the liver. Bile acids synthesized in the liver are secreted into the intestine, where they facilitate lipid absorption and experience wide alternation by the gut microbiota, shaping a dynamic bile acid pool that also works in metabolic and immune signaling [95]. Because peroxisomes are necessary for maturating bile acids, they assist in determining the composition of bile acids which enter the intestine [27,70]. In peroxisomal disorders, accumulation of C27 intermediates is caused by defective side-chain shortening, describing how the bile acid pool is shaped by peroxisomal function [27,96].

Disrupting this peroxisomal-regulated axis shows important implications for intestinal effects on liver disease. The microbiota and intestinal obstacle can be affected by changed bile acid composition. In virtue of receptor-mediated signaling pathways, epithelial integrity and immune reactions are controlled by bile acids, while bile acid activity is further changed by microbial metabolism [97,98,99]. Impaired obstacle integrity and grown intestinal and systemic inflammation have been related to imbalance in these processes [100]. These variations are especially related in chronic liver diseases. In ALD and associated circumstances, bile acid dysregulation is related to dysbiosis, obstacle dysfunction, and grown translocation of microbial products into the portal circulation, which amplifies hepatic inflammation [101,102]. On the contrary, recovery of bile acid homeostasis attenuates liver injury in experimental models while improving barrier function [103], backing the significance of bile acid-dependent exchange in disease progression.

An additional possibility is that peroxisomal dysfunction may influence the liver–gut axis through mature bile acids and accumulation of bile acid intermediates. The delivery of these intermediates to the intestine, where their unique physicochemical characters may change epithelial integrity or immune responses, could be increased by impaired peroxisomal processing. Although direct proof remains restricted, this idea emphasizes a potential effect for peroxisomes on regulating the quality of bile acid pool in inter-organ communication.

### 5.2. Role of Peroxisomes in the Liver–Kidney Axis: Metabolic Coupling and Systemic Stress

The liver and kidney are among the organs richest in peroxisomes, showing their shared effects on lipid metabolism, detoxification, and redox balance. This metabolic overlap is related to chronic liver disease, which is recognized more to have significant renal results. According to recent research, lipid dysregulation, oxidative stress, and inflammatory signaling are common drivers of injury in both organs, backing the idea of a metabolically coupled liver–kidney axis [104,105]. Clinical liver–kidney crosstalk is well established, especially in advanced cirrhosis where hepatorenal syndrome stands for a major complication. Experimental evidence further suggests that metabolic signals derived from liver injury may directly contribute to renal dysfunction. In a murine model of MASH, animals suffered from chronic kidney disease accompanied by renal transcriptomic variations enriched for lipid metabolism and fatty acid β-oxidation pathways [106]. Remarkably, renal function and regressed kidney injury were enhanced by transplantation of normal livers into MASH mice, suggesting that liver-derived metabolic signals can exert a direct impact on the kidney [107]. Despite incompletely defined specific contribution of peroxisomes to this axis, their central effect on controlling lipid and redox metabolism indicates a plausible link. Disturbances in hepatic metabolism may drive systemic stress by the circulation of lipid intermediates, oxidative imbalance, and inflammatory mediators, effecting renal function in turn. By comparing with the liver–gut axis, the effect of peroxisomes on liver–kidney communication remains less well established and demands further research.

### 5.3. Role of Peroxisomes in the Liver–Brain Axis: Lipid Metabolism and Neuroactive Signaling

Hepatic peroxisomal metabolism may also affect brain function, mainly by regulating the circulation of lipid species and bile acid profiles. Peroxisomes are necessary for the biosynthesis of docosahexaenoic acid and ether lipids, such as plasmalogens, which are basic ingredients of neuronal membranes and normal brain function [108]. Because the liver serves as a main source of circulating lipids, the availability of these metabolites to the brain could be affected by changes in hepatic peroxisomal function. Inherited peroxisomal disorders, in which hepatic and neurologic dysfunction are among the most obvious clinical manifestations, emphasize the close metabolic reliance of both the liver and brain on peroxisomal pathways [108]. Nevertheless, the specific interaction between hepatic peroxisomal dysfunction to liver–brain communication remains incompletely defined in chronic liver diseases.

In addition to lipid supply, a plausible signaling link between the diseased liver and the brain is offered by bile acids. Bile acids can affect neuroinflammatory and metabolic pathways in the central nervous system while crossing the blood–brain barrier [109,110]. More extensively, liver–brain exchange has been associated with bile acid dysregulation in chronic liver disease [111], indicating that brain signaling could be indirectly influenced by changed bile acid composition caused by hepatic peroxisomal dysfunction.

## 6. Therapeutic Opportunities and Translational Barriers

Recognition of peroxisomes as regulators of lipid composition, redox balance, and organelle communication raises the possibility that targeting peroxisomal pathways could provide therapeutic benefit across a range of liver diseases. Nevertheless, present measures mainly work indirectly by adjusting transcriptional programs or downstream metabolic pathways, and a main divide remains between mechanistic insight and useful clinical intervention. Therapeutic methods across various levels, from indirect modulation of peroxisome-associated pathways to novel measures designed to restore organelle function, are summarized in Table 1 and discussed below. Core obstacles to translation are further emphasized.

### 6.1. Targeting Transcriptional Programs: Promise and Limitations of PPAR Signaling

Peroxisome proliferator-activated receptors, especially PPARα and PPARγ, stand for the most widely researched measure for adjusting peroxisome-related pathways in liver diseases. Genes involved in fatty acid oxidation and oxidative metabolism are regulated by PPARα, whereas PPARγ keeps hepatic stellate cell quiescence and controls fibrogenic reactions. Therefore, pharmacologic activation of PPARs has been extensively explored as an indirect method to improve peroxisome-associated metabolic functions [112,116,117]. Experimental research offers uniform support for this method. Activating PPARα drives lipid clearance while suppressing inflammatory signaling in metabolic liver disease models [113,116]. In parallel, stellate cell activation and extracellular matrix generation are inhibited by PPARγ signaling; stellate cell activation is accompanied by loss of PPARγ, whereas fibrogenesis is suppressed by restoration of its activity [79,82,114]. Pharmacologic PPAR agonists, including pan-PPAR agents such as lanifibranor, have shown antifibrotic and anti-inflammatory roles in preclinical models and early clinical research, backing the relevance of this pathway [60].

The therapeutic relevance of PPAR signaling is further emphasized by recent clinical success in cholestatic liver disease. In primary biliary cholangitis (PBC), PPAR-targeting agents including seladelpar (PPARδ agonist) and elafibranor (PPARα/δ agonist) have displayed important efficacy and received FDA approval for patients with insufficient reaction to ursodeoxycholic acid [115,118,119]. In addition, cholestatic markers and clinical results in PBC are improved by fibrates including fenofibrate (PPARα agonist) and bezafibrate (pan-PPAR agonist), supporting a broader role for PPAR-directed metabolic modulation in chronic liver disease.

Nevertheless, clinical translation is still variable across disease contexts. Enhancements in lipid metabolism or inflammation do not uniformly bring obvious histological benefit, especially in advanced disease [60]. A core restriction may be that PPAR agonists work widely across metabolic pathways and organelles instead of directly recovering peroxisomal function. Hence, their effects are indirect and context-dependent, affected by disease stage, metabolic state, and species-specific diversities in receptor activity. According to this, decreased PPARα expression in metabolic liver disease has been related to impaired oxidative pathways, yet these defects may not be fully corrected by pharmacologic activation [113,117].

### 6.2. Modulating Lipid Composition and Metabolite Quality

An alternative measure is to target the downstream results of peroxisomal dysfunction by adjusting lipid composition and metabolite pools. Methods in this category encompass bile acid-based therapies, lipid-lowering agents, and interventions that change fatty acid flux [2,27].

In cholestatic liver disease, agents that adjust bile acid composition or signaling, such as FXR agonists, have shown clinical effect [97,121]. These therapies can partially compensate for changed bile acid metabolism by decreasing bile acid synthesis and/or driving clearance. Similarly, the burden on peroxisomal pathways in metabolic liver disease may be alleviated by interventions that decrease lipid overload or redirect lipid flux [116,120]. Nevertheless, these measures primarily solve downstream effects instead of the underlying defect in metabolite processing. Peroxisomes control the composition and quality of multiple lipid species in the meantime, and metabolic balance may not be fully restored by targeting a single pathway. Hence, these methods can mitigate disease progression but are unlikely to solve the wider defects in metabolite handling related to peroxisomal dysfunction.

### 6.3. Targeting Redox Balance and Oxidative Stress

Given the key effect of peroxisomes on hydrogen peroxide metabolism, adjustment of redox balance stands for another potential therapeutic avenue. Antioxidant therapies have been researched widely in liver disease, with the objective of decreasing oxidative stress and restricting tissue injury. In preclinical models, hepatocellular injury and inflammation can be attenuated by agents that decrease oxidative stress [123,125]. In clinical settings, antioxidants including vitamin E have displayed modest benefit in chosen patients with MASH, enhancing histological characteristics such as steatosis and inflammation [122,124].

While several agents display benefit in preclinical models, clinical results have been inconsistent. One restriction is that physiological redox signaling may be disrupted by global antioxidant measure, which is necessary for normal cellular function. Hence, physiological signaling pathways may be disrupted by suppressing oxidative stress widely without correcting the underlying metabolic imbalance. In addition, peroxisomal redox state is closely associated with metabolic flux, especially lipid oxidation, representing that excess oxidant generation often shows upstream disturbances in substrate handling instead of isolated defects in detoxification. Restoring coordination between oxidant generation and detoxification may be involved in a more useful method, instead of suppressing ROS globally. This could encompass improving peroxisomal antioxidant capacity or enhancing integration with mitochondrial redox systems, although such measures remain mainly experimental.

### 6.4. Restoring Organelle Function and Communication

An emerging strategy is to target peroxisomal dysfunction at the level of organelle integrity and inter-organelle communication, rather than individual metabolic pathways. As discussed, peroxisomes operate within a highly interconnected network involving mitochondria, ER, and lipid droplets, and disruption of this network contributes to metabolic imbalance and cellular stress [3,5]. Several approaches aim to restore peroxisomal function more directly. Enhancement of peroxisomal biogenesis through activation of transcriptional regulators such as peroxisome proliferator-activated receptor gamma coactivator 1 alpha (PGC-1α) has been shown to improve oxidative metabolism and organelle function in experimental systems [127,129,130]. Meanwhile, improving protein import into peroxisomes mediated by peroxin-dependent pathways represents a potential strategy to restore enzymatic activity, although this remains largely unexplored in liver disease. Maintenance of organelle quality is another key component. Selective turnover of damaged peroxisomes through pexophagy assists in preserving functional organelle populations and stopping accumulation of dysfunctional compartments [126,128]. In addition, membrane contact sites with mitochondria and ER are formed by peroxisomes. Adjusting these interplays may assist in restoring metabolic integration across organelles.

An ordinary merit is shared by them: they are designed to modify peroxisomal dysfunction at the dimension of organelle function and network coordination, instead of compensation for downstream metabolic results. Currently, these methods are in early stages of growth, and few have been applied to experimental systems. However, a conceptual merit is provided by solving peroxisomal dysfunction at the dimension of organelle integrity and network function, instead of separated metabolic endpoints.

## 7. Conclusions and Future Perspectives

Peroxisomes have served as core regulators of hepatic metabolism, exceeding fatty acid oxidation and reactive oxygen species detoxification. In hepatocytes, they govern metabolite quality, redox balance, and organelle integration, thereby shaping cellular stress reactions, inflammation, and tissue remodeling. Across liver diseases, peroxisomal dysfunction makes contributions to disease progression in virtue of uniform roles in lipid composition and metabolic signaling.

A key topic is that peroxisomes work as metabolic quality control mechanisms. In early stages of stress, they adapt to back lipid processing and redox balance, whereas continuous injury results in maladaptive states. This transition offers a structure for knowing disease progression from steatosis to inflammation, fibrosis, and malignancy. In addition, peroxisomes affect systemic physiology by regulating circulating metabolites that mediate liver–organ exchange. Changed bile acid composition may make contributions to extrahepatic injury and systemic inflammation, emphasizing the significance of metabolite quality in disease systems.

Although there are important advances, core difficulties still exist. There are no credible approaches to evaluate peroxisomal function in vivo, especially methods capturing dynamic metabolic flux. Clarification is demanded by the cell type-specific effect of peroxisomes in the liver and their interplay with other organelles. In addition, therapeutic measures remain mainly indirect, highlighting the demand for moving toward methods that recover peroxisomal function and uniform metabolite processing. Future efforts are required for paying attention to the definition of peroxisomal dynamics across disease stages, development of flux-based biomarkers, and recognition of measures that enhance organelle integration and metabolic balance. Pushing these regions forward will be key to changing peroxisomal biology to more accurate and useful interventions for liver disease.

## Figures and Tables

**Table 1:** Therapeutic strategies targeting peroxisomal pathways and functions in liver disease.

Strategy	Target Pathway	Representative Agents	Preclinical Models	Clinical Status	Refs.
Targeting transcriptional programs (PPAR signaling)	Indirect enhancement of peroxisomal fatty acid oxidation, anti-inflammatory signaling, and stellate cell quiescence.	Fenofibrate (PPARα), Bezafibrate (pan-PPAR), Seladelpar (PPARδ), Elafibranor (PPARα/δ), Lanifibranor (pan-PPAR)	Reduced steatosis, inflammation, and fibrosis in MASLD/MASH and fibrosis models; suppression of hepatic stellate cell activation	Seladelpar and Elafibranor approved for PBC; Lanifibranor in clinical development for MASH	[60,79,82,112,113,114,115,116,117,118,119]
Modulating lipid composition and metabolite quality	Restoration of bile acid homeostasis, reduction in lipid overload, and improvement of metabolite quality	FXR agonist, Ursodeoxycholic acid (UDCA), lipid-lowering and metabolic interventions	Improved bile acid metabolism, reduced fibrosis, alleviated liver injury in experimental MASLD/MASH and cholestatic disease models	UDCA is standard therapy for PBC; OCA approved for PBC and evaluated in MASH	[2,27,97,120,121]
Targeting redox balance and oxidative stress	Reduction in oxidative stress and restoration of oxidant-antioxidant balance	Vitamin E and other antioxidant compounds	Reduced hepatocellular injury and inflammation in experimental liver disease models	Modest benefit reported in MASH; inconsistent clinical efficacy overall	[122,123,124,125]
Restoring organelle function and communication	Enhancement of peroxisome biogenesis, protein import, and peroxisome-organelle crosstalk	PGC-1α activation strategies; experimental approaches targeting PEX proteins, pexophagy pathways, and organelle contact sites	Improved oxidative metabolism, organelle quality control, and metabolic integration in experimental systems	Largely preclinical; no established clinical therapies	[126,127,128,129,130]

## Data Availability

No new data were created or analyzed in this study.

## Abbreviations

ABCD3, ATP binding cassette subfamily D member 3; ACOX, acyl-CoA oxidase; ALD, alcohol-associated liver disease; CYP2E1, cytochrome P450 2E1; ER, endoplasmic reticulum; FXR, farnesoid X receptor; HCC, hepatocellular carcinoma; IL-1β, interleukin-1 beta; MASH, metabolic dysfunction-associated steatohepatitis; MASLD, metabolic dysfunction-associated steatotic liver disease; mTOR, mechanistic target of rapamycin; NAD^+^, nicotinamide adenine dinucleotide (oxidized form); NADH, nicotinamide adenine dinucleotide (reduced form); NF-κB, nuclear factor kappa-light-chain-enhancer of activated B; PBC, primary biliary cholangitis; PEX, peroxin; PGC-1α, peroxisome proliferator-activated receptor gamma coactivator 1 alpha; PPAR, peroxisome proliferator-activated receptor; ROS, reactive oxygen species; SIRT5, sirtuin 5; SMAD, suppressor of mothers against decapentaplegic; TGF-β1, transforming growth factor beta 1; TGR5, Takeda G protein-coupled receptor 5.
